# Improved Infant Outcomes with Group Prenatal Care in Puerto Rico

**Published:** 2017-02-06

**Authors:** Carmen D Zorrilla, Isalis Sánchez, Ana María Mosquera, Dianca Sierra, Lyz Annette López Pérez, Silvia Rabionet, Juana Rivera-Viñas

**Affiliations:** 1 Obstetrics and Gynecology Department, UPR School of Medicine, Puerto Rico; 2 Department of Human Genetics, Miller School of Medicine, University of Miami, USA; 3 Maternal Infant Studies Center (CEMI), Obstetrics and Gynecology Department, UPR School of Medicine, Puerto Rico

## Abstract

**Objectives:**

To evaluate the impact of group prenatal care (*Centering Pregnancy*) on the rate of Preterm Birth (PTB) and low birth weight. Women were enrolled into Centering Pregnancy (*Transformación Prenatal*) if they fell in the category of poverty, and had at least one risk for PTB according to known risk factors for low birth weight or PTB.

**Methods:**

Mother’s age, parity, risk factors, prenatal/delivery complications, infants’ Gestational Age (GA), birth weight, Apgar scores, delivery route, indications for delivery, and use of Neonatal Intensive Care Unit (NICU) were abstracted from charts of mothers who received group or traditional care at the University Hospital in San Juan, PR.

**Results:**

More infants were born at term if the mothers received *Centering Pregnancy*. The mean birth weight and gestational age of the infants were higher (6.59 vs. 6.33 lbs. and 37.8 vs. 36.8 weeks) than for those in traditional care. *Centering Pregnancy* also had lower rates of preterm birth (27.7% vs. 34.1%) and births earlier than 31 weeks (2.8% vs. 9.9%). All were statistically significant (P<0.05).

**Conclusions:**

We successfully implemented group prenatal care (*Centering Pregnancy*) for the first time in PR in a complex environment: tertiary care hospital with a high-risk prenatal clinic. Despite having known risk factors for preterm birth, the mothers in *Centering Pregnancy* had better outcomes. In an environment of adverse determinants of health, the program was effective in reducing the odds for adverse infant outcomes early in life and demonstrating that innovative models of health care can improve such outcomes.

## Introduction

Low birth weight is associated with increased morbidity and mortality in later life from conditions like cardiovascular disease, type 2 diabetes, and metabolic syndrome [1, 2]. Preterm birth (PTB- birth before 37 weeks) and related causes of death together accounted for 25% of all infant deaths in 2010 and in 2014 in the USA. The 10 leading causes of infant mortality in the USA accounted for 69.1% of the infant mortality in 2014. Two of them (short gestation and low birth weight, not elsewhere classified and maternal complications of pregnancy) accounted for 25% of the mortality for 2014 [3]. One of every 10 infants born in the USA in 2015 was preterm [4]. Large differences in risk of preterm birth remain for racial and ethnic groups. In 2014, black infants were about 50% more likely to be born preterm than white, Hispanic, and Asian/Pacific Islanders [5]. Preterm births accounted for $26 billion of expenses in health care in the USA [6]. The March of Dimes Premature Birth report cards show decreasing trends in the rates of PTB for the USA from 12.8% in 2006 to 11.7% in 2011 and to 9.6% in 2015 [7]. In the 2015 report for Puerto Rico, one of every 8 infants (11.8%) was born preterm [8]. In spite of decreasing trends of 19.9% in 2006, 17.6% in 2011 and 11.8% in 2013 the island is above the national rate and targets for PTB about 1.3 times that of the rate for the USA [9]. Birth rates have decreased by 24% from 52,239 in 2004 to 38,974 in 2014 [5]. The majority (65.4%) of the births in the island in 2012 were among unmarried women, adolescents comprised 17.1% of the mothers delivering that year (89% of them single) and about one in 6 overall had less than high school [9]. Poverty is one of the social determinants of health related to disparities and poor outcomes. The Puerto Rico poverty rate (45%) is much higher than the reported rates for the poorest states: Mississippi (28.9%) and New Mexico (26.4%) [10].

Additional factors related to poor outcomes include obesity and substance use such as cigarette and alcohol [11]. Obesity [12], and mental health issues [13] have been documented for youth in PR and the relevance to pregnancy outcomes is not proven yet, but might point to potential confounders.

Other social problems such as exposure to violence [14], public concerns with crime rates [15], high unemployment rate (PR 12% vs. 5% USA) [16,17], and high divorce rate [18] might also impact mental health and personal stress [19], which might affect the outcomes of pregnancy as well.

The proportion of women initiating prenatal care in the first trimester is similar in both (83% in the USA and 80% in PR) [9,20]. In spite of having access to prenatal care early in gestation there is still a high rate of preterm birth in PR. We propose that the content and quality of prenatal care as well as the prevalence of environmental and social factors are affecting the rates of PTB in the island. Group Prenatal Care known as Centering Pregnancy has been demonstrated to be effective to reduce the rate of preterm birth among pregnant women in the USA [21]. This manuscript will present the outcomes of *Centering Pregnancy* and its impact on reducing preterm birth rates in the target population.

## Materials and Methods

The Strong Start for Mothers and Newborns initiative, an effort by the Department of Health and Human Services, aimed to reduce preterm births and improve outcomes for newborns and pregnant women. The Centers for Medicare & Medicaid Services (CMS) funded projects that implemented specific prenatal care approaches to determine their efficacy in reducing preterm births among populations at high risk [22]. The goal was to determine whether these new approaches to care could increase the gestational age of neonates sufficiently to decrease the anticipated total cost of medical care over the first year of life for children born to high-risk mothers.

This is a report of the comparison of the outcomes of the mothers enrolled in *Centering Pregnancy* at the University Hospital in San Juan, PR funded with this initiative and the outcomes of women in Traditional Care (individual one-on-one visits with medical providers, in this case Ob-Gyn residents and faculty). The program started on August 2013. We titled our initiative: *Transformaciòn Prenatal* (TP), which means prenatal transformation and is a direct description of our target with the health care delivery model and with the patients.

*Centering Pregnancy* (TP) was provided to women if they had at least one risk for preterm birth per the known risk factors for low birth weight or preterm labor (List on Table 1) and enrolled in the public-funded health system titled *PR Mi Salud* (equivalent to Medicaid). All women presented to prenatal care with a gestational age of less than 24 weeks from August 2013. Later we were allowed to enroll women who presented to care up to 29 weeks GA (as of June 2015). The University Hospital is a referral hospital for most high-risk pregnant women in the island with about 35% of the infants born in 2012 weighting less than 2,500 g (unpublished data). Women who presented later in pregnancy were not entered *Centering Pregnancy*. These might have presented some bias, which we discuss at the end of this document.

As for any service offered at the hospital, written consents are taken for receiving care and for participation into the groups. The consents are approved by the Hospital Records Department. The protocol was approved by the University of Puerto Rico Medical Sciences Campus (UPR-MSC) Institutional Review Board (IRB protocol number: 1350115). Table 1 lists the documented risk factors for preterm birth [23,24]. This list was used as part of the assessment of women to be offered participation in the care model of group prenatal care.

This analysis includes the clinical outcomes of the patients enrolled during the first two years of the program. Data was obtained from the Labor Room records of patients who delivered at the University Hospital between August 2013 and December 2015. A total of 1,726 records were reviewed, of those 614 corresponded to women receiving *Centering Pregnancy* (TP) and 1,112 receiving traditional care. We obtained information on all women (N=1,726) delivering at the hospital for the target period.

We obtained Infants’ date of birth, Gestational age (GA), mother’s age, parity, diagnosis and prenatal/delivery complications, infant’s weight, Apgar scores at 1 and 5 minutes, and complications from the delivery records. Delivery route (vaginal or Cesarean Section-C/S), indications for delivery (i.e. emergency C/S, labor induction, vaginal birth after a cesarean-VBAC and others), and the need for transfer to neonatal intensive care unit (NICU) was also abstracted. Patients were classified as having received group or traditional prenatal care.

### *Centering Pregnancy* Care Model

According to the Centering Healthcare Institute “the *Centering Pregnancy* model is a group model that utilizes a facilitative leadership style that honors basic principles of adult education. The content that is found in traditional childbirth classes is woven throughout the ten *Centering Pregnancy*. It’s a model of group health care, which incorporates three major components: assessment, education, and support. Group participants meet with their care provider according to a regular schedule for a much longer period of time (usually 90–120 minutes) than a traditional visit. Women with similar gestational ages meet, learning care skills, participating in a facilitated discussion, and developing a support network with other group members (8–12 participants per group). Each group meets for a total of 10 sessions throughout pregnancy. The practitioner, within the group space, completes standard physical health assessments. The women learn as much or more from other women in the group which contributes directly to an increasing sense of empowerment [25]”.

As of December 2015 a total of 877 women had been enrolled in the program: 70% unemployed, 22% married, and 65% in a relationship, 5% with pre-pregnancy Type 1 DM, 10% with pre-pregnancy Type 2 DM, and 14% with prior diagnosis of Hypertension. Among the current pregnancy complications 9% had gestational Diabetes, and 7% hypertension.

### Program Implementation

The program was implemented late in 2013 to a sub-group of patients with high-risk. It was expanded to the majority of patients entering prenatal care prior to 29 weeks GA with a known risk factor. Many challenges were confronted including space, staffing, training, coordination and scheduling of the groups, evaluation of the outcomes and patient satisfaction, patient needs and recruitment, and planning for sustainability.

The group started with staff training in the centering model of care and the preparation of the infrastructure for the group sessions. The Hospital provided one dedicated room, which was prepared with all of the requirements to run the sessions (an exam table on a private corner, chairs arranged in a circle, a small fridge for refreshments, desks and tables for materials). The room for the sessions followed the guidelines for Centering Pregnancy established by the Centering Healthcare Institute (CHI) and staff from CHI provided the initial training [26]. Our group will seek certification of the program once the implementation is complete and data and additional outcomes are available.

We confronted many challenges in order to implement the program, which have included resistance to change from current personnel at the clinics (appointments, billing, nursing, and other services). Changing a culture is difficult, especially when we want to empower patients, have a system that is more available and friendly to patients and partners, and that improves efficiency. The traditional model contemplates long lines of patients waiting for services instead of a flow of less patients waiting. Our goal was to enroll all pregnant women presenting for prenatal care at or below the 24^th^ gestational age. In spite of having eligible patients seen at the clinics, we were not recruiting as many patients as were available. During the first year we only reached one third of the potential participants. During the initial year both models of care were provided in the clinic since we were developing the expertise needed to implement the model in the whole clinic at a later time. This duality of service models did not allow for the full participation of the staff and the enrollment of all the women who could have benefited. To increase the capacity we identified second room prepared so that simultaneous sessions could be carried out. In addition, several meetings were held with the clinic staff and the policy of the group care as the universal model of care was established. So, after the first year, the whole clinic provided care with the *Centering Pregnancy* care model.

### Statistical Analysis

Overall descriptive statistical analyses, and within each group were obtained in order to describe the socio-demographic characteristics, and pregnancy outcomes of the groups studied. Frequencies and percentages were calculated for categorical variables and central tendency and statistical dispersion measures were obtained for continuous variables. Also, differences between groups (traditional vs*. Centering Pregnancy*) were analyzed using Chi-square analysis for categorical variables and one-way ANOVA for continuous variables.

## Results

The overall sample consisted of 1,726 women who delivered at the University Hospital of which 614 received *Centering Pregnancy* care and the other 1,112 received traditional prenatal care. The overall mean age was 26.65 years and the mean Gestational Age (GA) at delivery was 37.20 weeks. On the other hand, the mean birth weight of infants was 6.43 lbs. (2,915.83gm). Almost half (47.9%) of the deliveries were through a Cesarean (C/S). One fourth of the infants (25.7%) was transferred to the neonatal intensive care unit (NICU) either due to low birth weight or needs for special care.

### Outcomes of *Centering Pregnancy* vs. Traditional Care

The mean age of the pregnant women was similar (26.15 for the *Centering Pregnancy* and 26.70 for the traditional care). The age ranges were from 13 to 49 years of age. The mean birth weight of the infants for group care was higher (6.59 vs. 6.33 lbs.), as well as the mean GA at delivery (37.83 vs. 36.85 weeks). Infants had higher Apgar scores at 1 and 5 minutes in the *Centering Pregnancy* (7.69 vs. 7.23 and 8.66 vs. 8.38). The differences in birth weight, gestational age at birth Apgar scores and proportion of preterm deliveries were all statistically significant (Table 2). The rate of preterm delivery (less than 37 weeks GA) was 34.1% for women receiving traditional care. The rate of preterm delivery (less than 37 weeks GA) was 27.7% for women receiving *Centering Pregnancy*. This was statistically significant. The differences in PTB were also significant for those born less than 31 weeks (2.8% vs. 9.9%) for *Centering Pregnancy* vs. traditional care.

## Discussion

There are several known reported risk factors for PTB including: previous preterm birth, multiple gestation, cervical incompetence [15], maternal chronic disease such as hypertension and diabetes [27], infections, cigarette smoking, alcohol use, or illegal drug use during pregnancy among others [15,24]. Many of these factors have been listed as social determinants of health including poverty [24], lack of education, lack or difficulty with access to health care [28] and prevention strategies, personal and social exposure to violence, among others. The field is full of references and analysis supporting the impact of those factors on health outcomes [29].

Even though prenatal care provides a protective effect in reducing PTB, there are racial disparities that cannot be explained only on the basis of prenatal care. Vintzileos et al. [28], nevertheless conclude that the presence of prenatal care in the USA, seems to be related to lower PTB in populations with and without high risk conditions. To improve health outcomes such as preterm birth and low birth weight, several strategies and policies might be needed in addition to making accessible prenatal care.

Among the interventions with demonstrated efficacy to reduce preterm birth are the progesterone supplementation approved by the FDA on 2011 for women with a history of at least one prior spontaneous preterm delivery [29], and to a certain extent *Centering Pregnancy* care [21,30]. Ickovics et al. [30] documented the efficacy of the care model in reducing the rate of PTB. Birth weight was greater for infants of women Centering Pregnancy vs. individual prenatal care in a matched cohort study at public clinics comparing Centering with traditional care. A subsequent randomized trial comparing centering with traditional care and demonstrating equal or improved perinatal outcomes with no additional costs. Women in *Centering Pregnancy* had lower PTB rate compared with those in traditional care: 27.9% vs. 37.9%. This represented a risk reduction of 33%. Women *Centering Pregnancy* sessions had better prenatal knowledge and better satisfaction with care [21].

With the aim of improving outcomes of birth (which might improve later life health parameters), we implemented a program of Centering Pregnancy (Group Prenatal Care) among patients receiving care at a high-risk clinic in San Juan PR. This model of care has resulted in improvements in outcomes in the USA [21,30], therefore we decided to implement this model of care for the first time in the island and within a high-risk population. If early outcomes such as birth weight and gestational age at birth can be improved, we would not only be improving survival and quality of life, but perhaps affecting the risks for other morbidities later in life. This would be in fact a small step in addressing social determinants of health at early life.

Since we have previously reported group interventions for women living with HIV as part of an Empowerment model we chose to implement group prenatal care in our Hospital [31,32].

Our population includes the women referred for prenatal care at the University Hospital (UH) in San Juan, PR, which is the main tertiary care facility in the island and reports 1,200 deliveries per year and a high rate of LBW and preterm births. The University Hospital is a referral hospital for most high risk pregnant women in the island with about 35% of the infants born in 2012 weighting less than 2,500 g (unpublished data). This high risk prenatal clinic receives patients of all over the island and many of those with no medical insurance. The program is embedded in an accredited Ob-gyn residency program. As many programs in the USA, faculty is composed of generalists and subspecialists including Maternal Fetal Medicine (MFM). Therefore, patients with recognized risk factors for eligible interventions such as 17-OH progesterone (17-OH-P) are offered such interventions. The outcomes observed after the program was implemented are compared with the outcomes of women receiving traditional care before and during the implementation of the program. All interventions for reduction of PTB were available to both groups.

Improving the outcomes on this group of women and their infants is expected to have a greater impact on the islands’ overall birth outcomes since this group contributes to a large proportion of the preterm and low birth weight infants in PR.

The impact of low birth weight and preterm delivery affects the survival and morbidity of at risk populations. In addition, low birth weight has been associated to increased morbidity and mortality in later life from cardiovascular disease, type 2 diabetes, and the metabolic syndrome. Barker’s theory of “fetal programming” has used diverse epidemiological evidence to substantiate adult morbidity risks based on birth weight [1]. Improving birth weight will not only have individual benefits such as improved survival and better quality of life, but also will improve long-term outcomes and reduce risks of adult chronic illnesses as well. Improving the birth weight will decrease the cost of NICU for the populations in question since NICU costs are directly related to gestational age at birth and birth weight. The lower the birth weight, the higher the cost and length of stay [6].

Our program was successful in implementing Centering Pregnancy (group prenatal care) for the first time in Puerto Rico in such a complex environment: tertiary care hospital with a high-risk clinic and a training program. To our knowledge this is also the first primary Spanish-language program outside the continental USA. When compared with women who received traditional prenatal care (individual visits) with varying educational contents, the outcomes of the infants of women enrolled in *Centering Pregnancy* demonstrated higher gestational age at birth, increased birth weights and Apgar scores and a lower proportion of preterm birth at any of the different categories.

## Limitations

Among the limitations of this report is the retrospective nature of the design. All the women *Centering Pregnancy* were followed at the University Hospital clinics. Most of the women in traditional care were also cared at the University Hospital but some might have been referred from other providers due to pregnancy complications. This could present a selection bias towards more PTB in traditional care, since some might have been referred due to preterm labor.

Nevertheless, all the women not receiving *Centering Pregnancy* received traditional care, which is the variable of interest. In contrast, all the patients enrolled into *Centering Pregnancy* had at least one risk factor for preterm birth (PTB), which might have biased the group towards more PTB. Despite this potential bias in enrollment, the outcomes of the women *Centering Pregnancy* were significantly better. The selective enrollment of women with risk for PTB could have overcome the potential bias of referrals of women with preterm labor from the community.

Finally, if a program of *Centering Pregnancy* can be successfully implemented in a setting with high-risk populations and large number of providers, this model could be implemented in other settings in the island as well. The ultimate outcome could be the improvement in maternal and infant health and the reductions in morbidity associated to preterm or low birth weight.

## Figures and Tables

**Table 1:** Risk Factors for Preterm Birth ACOG [23,24].

History	Lifestyle Factors
Prior preterm birth	Low pre-pregnancy weight
Short cervical length	Obesity
History of cervical or uterine surgery	Smoking
Short interval between pregnancies	Substance abuse
**Pregnancy Complications**	**Other Factors**
Multiple pregnancy	Age < 17 years or > 35 years
Vaginal bleeding	African American race
Infections	Low socioeconomic status
Surgical procedures	Stress or anxiety disorders
Placental abnormalities	
Periodontal disease	
Fetal anomalies	
Abnormalities in fetal growth	
Diabetes mellitus	

**Table 2:** Outcomes of infants according mothers prenatal care (Centering Pregnancy vs. Traditional care)

Variable	*Centering Pregnancy* n=614	Traditional Care n=1,112	P-value*
Birth weight (BW) mean in gm.	2,990.6	2,871.7	0.002
≤ 2,500 gm. (%)	20.1	25.6	0.011
< 1,500 gm. (%)	2.8	7.6	0.000
Gestational Age (GA)	37.8	36.9	0.000
≤ 31 weeks GA (%)	2.8	9.9	0.000
≤ 36 weeks GA (%)	17.9	27.2	0.000
≥ 37.01 weeks GA (%)	72.3	65.9	0.006
Apgar Score 1 min	7.7	7.2	0.000
Apgar Score 5 min	8.7	8.4	0.000

* One-way ANOVA and Chi-square analysis were performed
